# Supplementary material for: Construction of a synthetic metabolic pathway for biosynthesis of the non-natural methionine precursor 2,4-dihydroxybutyric acid
Source: Nat Commun. 2017 Jun 20;8:15828. doi: 10.1038/ncomms15828 (PMC5481828; doi:10.1038/ncomms15828)
Supplement: Supplementary Information [file ncomms15828-s1.pdf]

Type of file: PDF
Size of file: 0 KB
Title of file for HTML: Supplementary Information
Description: Supplementary figures, supplementary tables, supplementary notes and supplementary references.

Type of file: PDF
Size of file: 0 KB
Title of file for HTML: Peer review file
Description:

Supplementary Figure 1: Optimal carbon flux distribution for the production of 2,4-dihydroxybutyric acid (DHB) in the central metabolic network of *E. coli*.

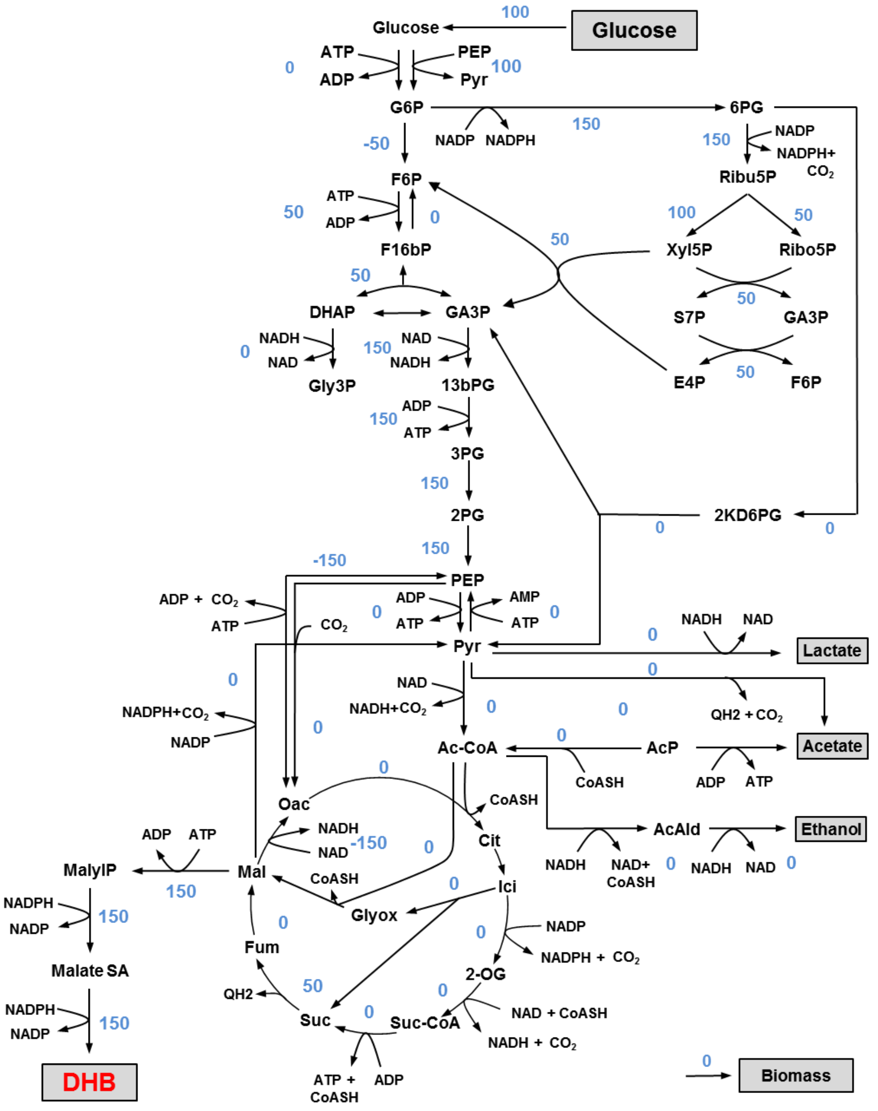

Fluxes are indicated in blue as mole percent per consumed glucose.(Abbreviations: MalylP – malyl phosphate, Malate SA – malate semialdehyde, G6P – glucose-6P, F6P – fructose-6P, F16bP – fructose-1,6-bisP, DHAP – dihydroxyacetonephosphate, GA3P – glyceraldehyde-3P, Gly3P - glycerol-3P, 13bPG – 1,3-bisphosphoglycerate, 2-phosphoglycerate, 3PG – 3P-glycerate, PEP – phosphoenolpyruvate, Ac-CoA – acetyl-CoA, AcP – acetyl-P, AcAld – acetaldehyde, Cit – citrate, Ici – isocitrate, 2-OG – 2-oxoglutarate, Suc-CoA – succinyl-CoA, Suc – succinate, Fum – fumarate, Mal – malate, Glyox – glyoxalate, 6PG – 6P-gluconate, Ribu5P – ribulose-5P, Ribo5P – ribose-5P, S7P – sedoheptulose-7P, Xyl5P – xylulose-5P, E4P – erythrose-4P).

Supplementary Figure 2: Active-site region in X-ray crystallographic structure of *E. coli* aspartate kinase III (Ec-LysC) ternary complex with Mg-ADP and (L)-aspartate in the R-state[10].

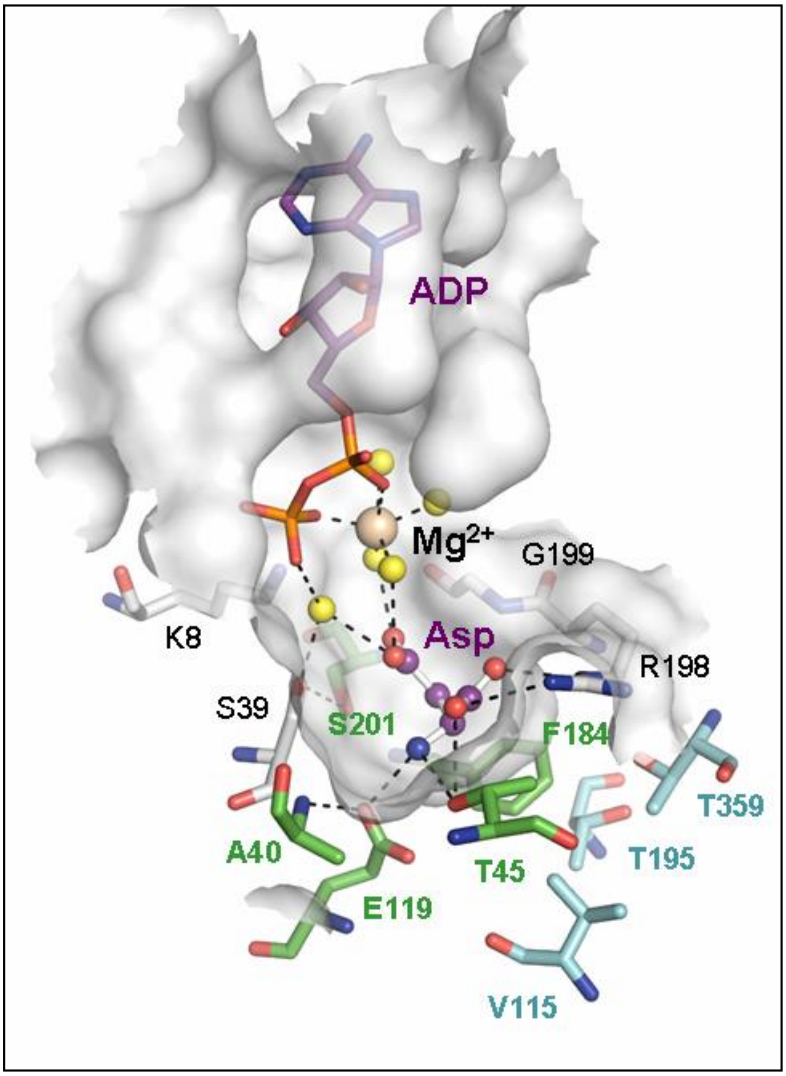

Carbon atoms in residue positions chosen for incorporation in the combinatorial library for experimental screening of malate kinase activity (Supplementary Table 5) are coloured in green (or cyan) according to whether (or not) direct residue contact is made with the (L)-aspartate substrate. Carbons in ADP and (L)-aspartate are coloured purple, and those in remaining enzyme residues are shown in grey. All other atoms are coloured according to element type: nitrogen, blue; oxygen, red; and phosphorus, orange. Bound water molecules are depicted as solid yellow spheres. Hydrogen bond interactions are indicated as inter-atomic dashed-line vectors. A diffuse molecular surface representation of the enzyme active-site is shown in grey. Binding of the (L)-aspartate substrate is orientated by water-mediated interactions of the β-carboxylate group with the metal ion (ochre coloured space filling sphere) of the Mg-ADP co-substrate, and salt bridge electrostatic interactions of the α-amino and α-carboxylate groups with charged Glu119 and Arg198 enzyme side-chains, respectively. The Thr45 side-chain can make additional hydrogen bonds to the (L)-aspartate α-amino and α-carboxylate groups. Other active-site residues in direct contact with the natural substrate include Phe184, lining the bottom of the active-site pocket, and Ser201 which hydrogen bonds to the β-carboxylate group of (L)-aspartate. The side-chain of Ser39 makes a metal co-ordinating water mediated hydrogen bond interaction with the β-carboxylate group of the substrate.

Supplementary Figure 3: Residue type counts at nine positions in 30,000 computationally designed Ec-LysC mutant complexes with (L)-malate.

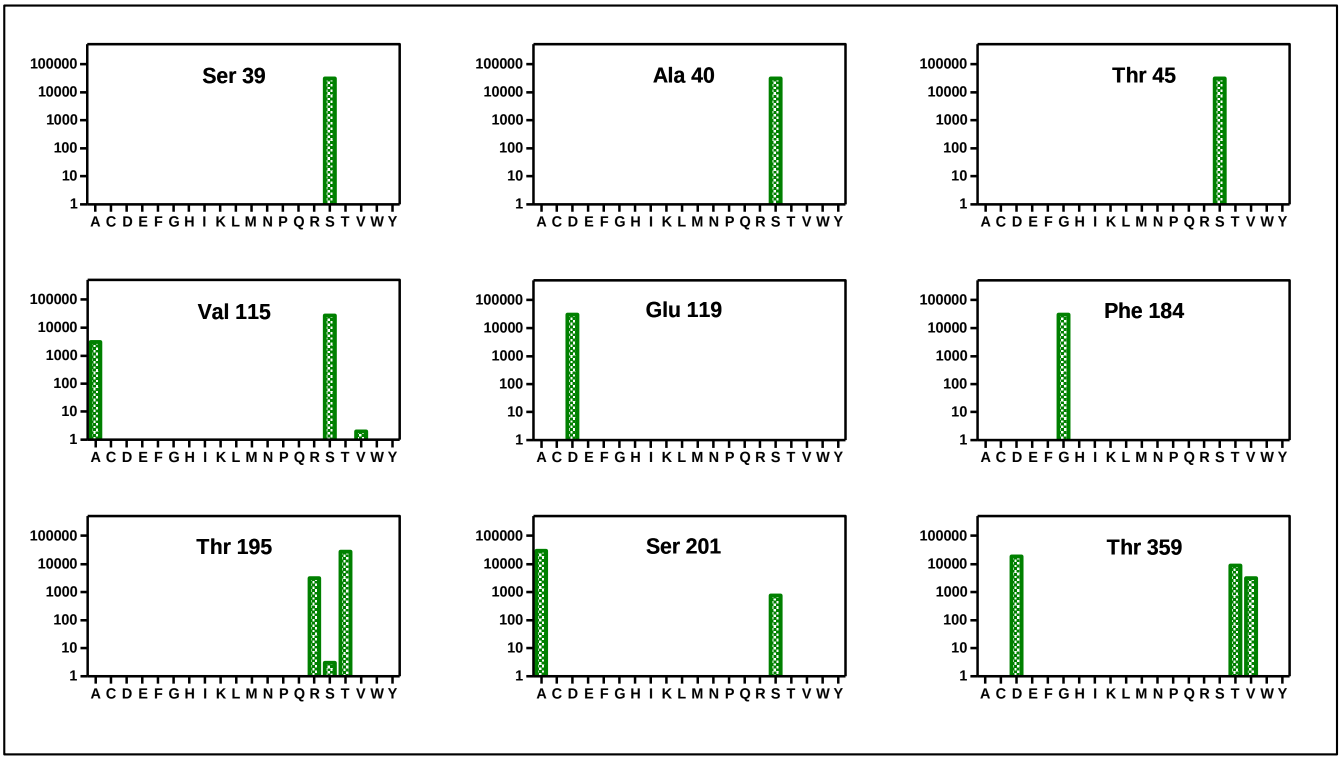

Supplementary Figure 4: Impact of selected mutations on the resistance of the malate kinase mutants LysC E119G and LysC V115A:E119S:E434V to increased lysine concentration.

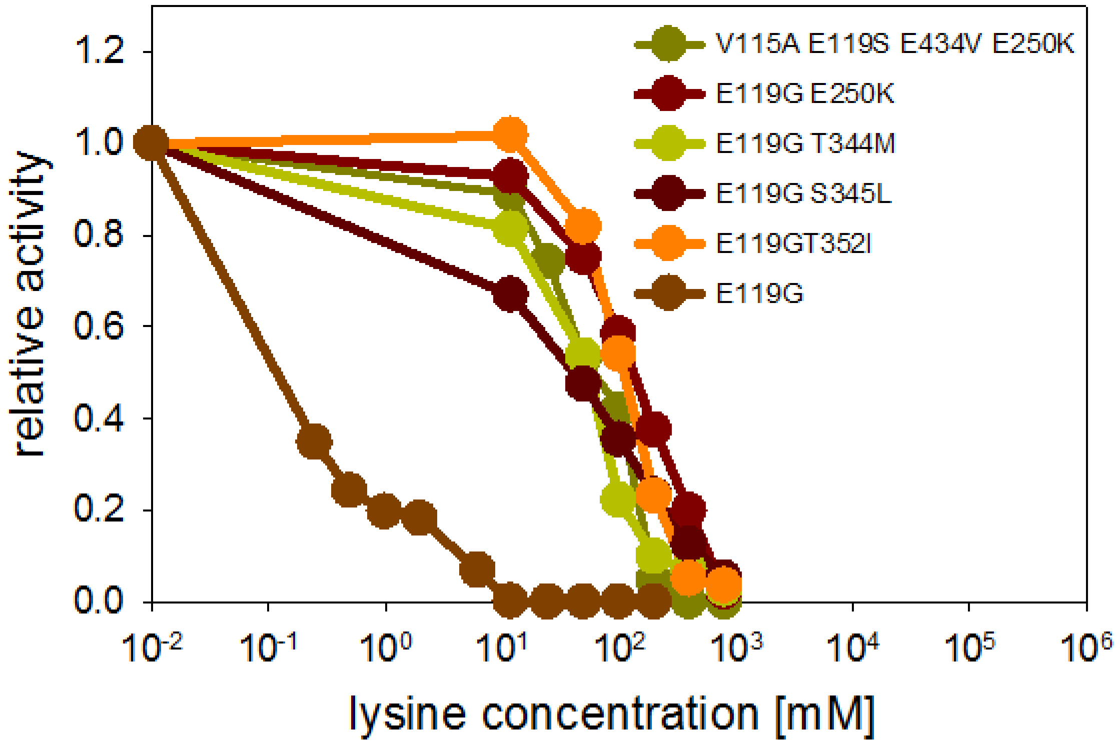

Supplementary Figure 5: Specific activities of aspartate semialdehyde dehydrogenase (Ec-Asd) mutants in position Glu241 on malate semialdehyde.

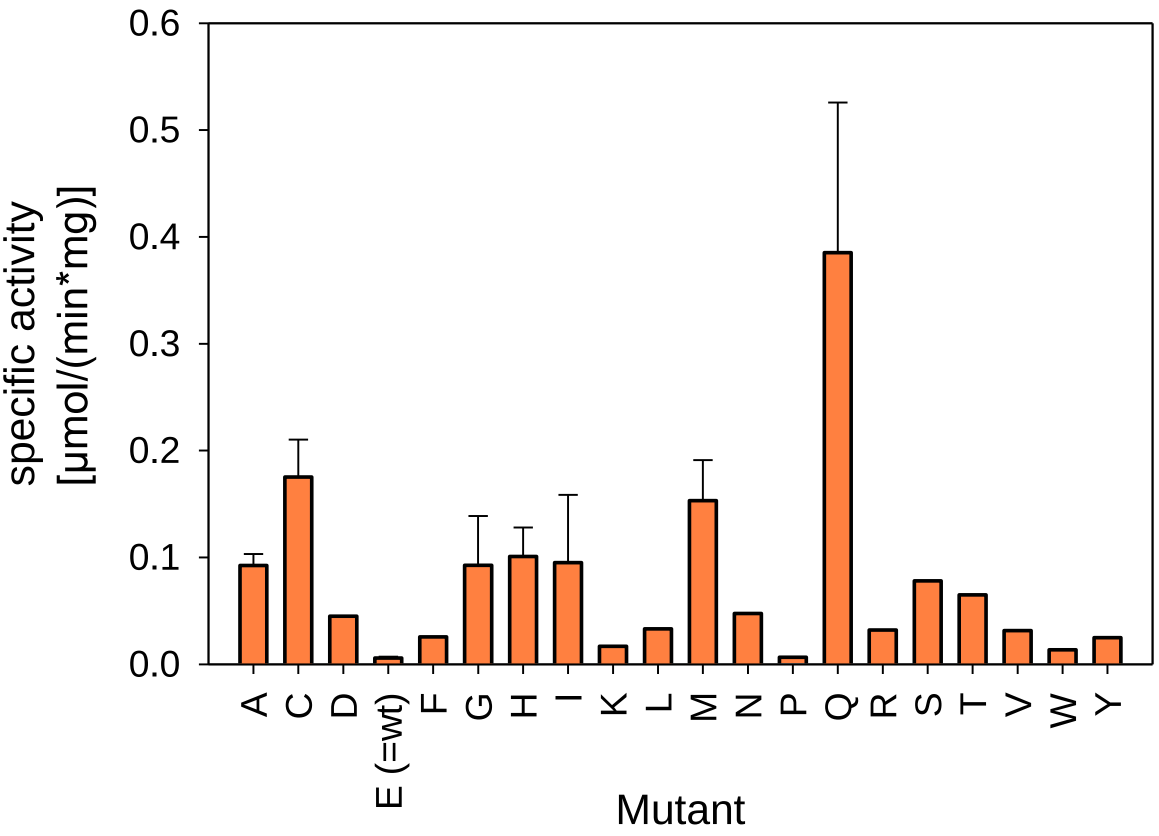

Data presented are the mean of at least two biological replicate experiments. Error bars correspond to the standard deviation of the mean.

Supplementary Table 1: Gibbs free energy of formation of DHB pathway intermediates

Compound	Contributing groups	$\Delta_f G^{0\prime}$ [kJ mol^{-1}]
malate^{2-}	2 x –COO^{1-}	-840.98
	1 x -OH	
	1 x -CH_2-	
	1 x –CH<	
malyl-P^{2-}	1 x –COO^{1-}	-1740.1
	1 x -OH	
	1 x -CH_2-	
	1 x –CH<	
	1 x -CO-OPO_3H^-	
malate-SA$^-$	1 x -COO^{1-}	-620.5
	1 x –OH	
	1 x -COH	
	1 x -CH_2-	
	1 x –CH<	
DHB$^-$	1 x -COO^{1-}	-660.2
	2 x –OH	
	2 x -CH_2-	
	1 x –CH<	
ATP^{3-}		-2819.4
ADP^{2-}		-1949.1
NADPH		-3082.9
$NADP^+$		-3104.9
HPO_4^{2-}		-1096.1
H^+		-39.9

Supplementary Table 2: Kinetic parameters of wild-type homoserine pathway enzymes on their natural substrates and the corresponding intermediates of the synthetic DHB pathway

	Natural substrate*			Synthetic substrate**		
Enzyme	k_{cat} [s^{-1}]	Km [mM]	K_{cat}/Km [s^{-1} mM^{-1}]	k_{cat} [s^{-1}]	Km [mM]	k_{cat}/Km [s^{-1} mM^{-1}]
Ec-LysC	13.2 ±0.4	7.9 ±0.2	1.7 ±0.1	0	-	-
Ec-Asd	36 ±12	-***		0.13 ±0.017	-***	-
Sc-Hom6	126 ±23	0.22 ±0.04	573	0	-	-

Ec – E. coli, Sc – S. cerevisiae, Enzyme (natural substrate*/synthetic substrate**): Ec-LysC – aspartate kinase III (aspartate/malate), Ec-Asd – aspartate semialdehyde dehydrogenase (aspartyl phosphate/malyl phosphate), Hom6 – homoserine dehydrogenase (aspartate semialdehyde/malate semialdehyde). (***) The Km values for Ec-Asd on aspartyl-P and malyl-P were not estimated because both substrates are highly unstable. Both substrates were provided by coupling the enzymatic test to aspartate or malate kinase. Data is presented as mean ± SD of at least two replicate experiments.

Supplementary Table 3: Kinetic parameters for wild type and reconstructed Ec-LysC mutants on (L)-malate and (L)-aspartate

Enzyme	(L)-Malate k_{cat} [s^{-1}]	(L)-Malate Km [mM]	(L)-Malate k_{cat}/Km [s^{-1} mM^{-1}]	(L)-Aspartate k_{cat} [s^{-1}]	(L)-Aspartate Km [mM]	(L)-Aspartate k_{cat}/Km [s^{-1} mM^{-1}]	Specificity*
WT	0	-	-	13.2 ±0.4	7.9 ±0.24	1.678 ±0.07	0
V115A:E119S:E434V	6.8 ±0.1	8.3 ±0.5	0.82 ±0.05	1.14 ±0.2	119.5 ±29.8	0.010 ±0.003	86
E119S	2.2 ±0.1	12.7 ±1.1	0.17 ±0.01	1.1±0.1	491.7 ±24.3	0.002 ±0.000	76
E119C	4.4 ±0.2	19.2 ±1.7	0.23 ±0.02	na	na	na	na
E119A	2.5 ±0.1	10.5 ±1.0	0.24 ±0.03	na	na	na	na
E119G	1.4 ±0.1	10.9 ±0.4	0.14 ±0.01	0.1 ±0.0	32.3 ±6.5	0.003±0.001	41
E119P	0.6 ±0,1	18.5 ±9.1	0.04 ±0.02	na	na	na	na
E119T	0.4 ±0,1	12.3 ±0,4	0.03 ±0.01	na	na	na	na
E119V	0.4 ±0.1	23.0 ±2.8	0.02 ±0.01	na	na	na	na
E119N	0.1 ±0.03	43.0 ±4.2	0.003 ±0.00	na	na	na	na
E119Q	0.03 ±0.004	38.5 ±0.7	0.001 ±0.00	na	na	na	na

na: Enzyme activity not analysed on (L)-aspartate. (*) Enzyme specificity is given by $(k_{cat}/Km)_{malate}/(k_{cat}/Km)_{aspartate}$. Kinetic parameter data is presented as mean ± SD of at least two independent replicate experiments. No detectable (L)-malate kinase activity was observed in Ec-LysC mono-mutants containing phenylalanine, tyrosine, tryptophan, histidine, methionine, leucine, isoleucine, lysine or arginine substitutions for glutamate at position 119.

Supplementary Table 4: Position-dependent residue type conservation and normalised Shannon entropy (H_x) at active-site region residue positions in a multiple sequence alignment of Ec-LysC homologues

Residue Type in Ec-LysC-	Residue Position	Substrate Binding-Site Shell No	Conservation of Ec-LysC Residue Type [%]	Entropy (H_X)
Ser	39[(a)]	1	83.5	0.33
Ala	40[(a,b)]	1	85.0	0.33
Thr	45[(a,b)]	1	99.7	0.02
Glu	119[(a,b)]	1	100.0	0.00
Phe	184[(a,b)]	1	93.7	0.09
Arg	198	1	99.5	0.01
Ser	201[(a,b)]	1	95.2	0.08
Val	115[(a,b)]	2	37.3	0.57
Thr	195[(a,b)]	2	56.8	0.47
Thr	359[(a,b)]	2	20.7	0.82

(a) Residue positions targeted in computational redesign (Supplementary Figure 3). (b) Residue positions included in experimentally screened combinatorial library (see Supplementary Table 5 below).

Supplementary Table 5: Allowed mutations in Ec-LysC active –site region combinatorial library

Residue Type and Position in Ec-LysC	Allowed Residue Types in Combinatorial Library	No. of Combinations
Ala 40	Ala, Ser	x2
Thr 45	Thr, Ser	x2
Val 115	Val, Ala, Ser	x3
Glu 119	Gly, Ser, Asn, Asp, Gln	x5
Phe 184	Phe, Gly	x2
Thr 195	Thr, Arg, Ser	x3
Ser 201	Ser, Ala	x2
Thr 359	Thr, Asp, Val	x3
	**Total:**	**2160**

Supplementary Table 6: Kinetic parameters of wild-type and mutant aspartate semialdehyde dehydrogenase enzymes on aspartyl-P (AP) and malyl-P (MP)

**Enzyme**	**$k_{cat\ (AP)}$ [s^{-1}]**	**$k_{cat\ (MP)}$ [s^{-1}]**	**Specificity ($k_{cat\ (AP)}/k_{cat\ (MP)}$)**
Ec ASD wt	36.2 ±12.23	0.13 ±0.017	285.8 ±104.6
Ec ASD E241Q	0.33 ±0.08	0.08 ±0.013	4.0 ±1.1
Ec ASD E241C	0.15 ±0.03	0.01 ±0.003	16.4 ±7.5
Bs ASD wt	14.45 ±6.13	0.04 ±0.003	327.5 ±140.9
Bs ASD E218Q	0.15 ±0.03	0.31 ±0.051	0.5 ±0.1
Bs ASD E218C	0.13 ±0.01	0.07 ±0.005	1.9 ±0.2
Mj ASD wt	0.38 ±0.09	0.04 ±0.005	9.9 ±4.5
Mj ASD E210Q	0.09 ±0.01	0.08 ±0.010	1.2 ±0.2
Mj ASD E210C	0.09 ±0.01	0.11 ±0.015	0.9 ±0.2

Ec – Escherichia coli, Bs – Bacillus subtilis, Mj – Methanocaldococcus jannaschii, Data represent the mean ± SD of at least two biological replicate experiments.

Supplementary Table 7: Activity of candidate aldehyde reductases on malate semialdehyde

Enzyme*	Natural substrate	EC number	Activity on MSA** [µmol/(min mg)]	Km on MSA [mM]	k_{cat}/Km [s^{-1} mM^{-1}]
Sc-Hom6	aspartate semialdehyde	1.1.1.3	No	-	-
Ec-GarR	tartronyl semialdehyde	1.1.1.60	No	-	-
Ec-GlxR	tartronyl semialdehyde	1.1.1.-	No	-	-
Ec-YihU	3-sulfolactaldehyde, 4-hydroxybutyrate	1.1.1.61	No	-	-
Ec-YqhD	manyaldehydes	1.1.1.2	1.2 ±0.36	ns	-
Ms-Ssr	succinic semialdehyde	1.1.1.B47	4.0 ±1.3	1.1 ±0.25	5.1
Pg-4hbd	succinic semialdehyde	1.1.1.61	3.6 ±1.3	5.1 ±0.1	0.98
Sc-Adh6	medium chain aldehydes	1.1.1.2	No	-	-
Sc-Ypr1	methylbutyraldehyde	1.1.1.-	0.19 ±0.05	ns	-

(*) Sc–*Saccharomyces cerevisiae,* Ec– *Escherichia coli,* Ms – *Metallosphaera sedula,* Pg – *Porphyromonas gingivalis.* (**) MSA = malate semialdehyde. Activity was estimated at a concentration of 20 mM MSA. ns – no saturation at substrate concentrations of up to 20 mM. Data represent the mean ± Std Dev of at least two biological replicate experiments.

Supplementary Table 8: Kinetic parameters for succinic semialdehyde reductase mutants from *Metallosphaera sedula* on succinic semialdehyde and malic semialdehyde

	Succinic semialdehyde			Malic semialdehyde			
Enzyme variant	k_{cat} [s^{-1}]	Km [mM]	k_{cat}/Km [s^{-1} mM^{-1}]	k_{cat} [s^{-1}]	Km [mM]	k_{cat}/Km [s^{-1} mM^{-1}]	Specificity*
WT	12.5 ±1.8	0.10 ±0.00	125 ±19	5.6 ±1.8	1.1 ±0.3	5.0 ±2.0	0.039 ±0.016
H39R	5.1 ±0.2	0.05 ±0.01	97 ±25	2.3 ±0.1	0.3 ±0.0	8.2 ±1.2	0.084 ±0.024
N43H	20.4 ±0.5	0.14 ±0.00	146 ±4	5.3 ±1.2	1.2 ±0.1	4.4 ±1.1	0.030 ±0.007
H39R:N43H	12.3 ±0.5	0.05 ±0.01	249 ±46	6.3 ±0.4	0.3 ±0.1	22.3 ±6.6	0.089 ±0.031

(*) Specificity is defined as the ratio of k_{cat}/Km on malic semialdehyde and k_{cat}/Km on succinic semialdehyde. Data are presented as the mean and SD from at least two biological replicate experiments.

Supplementary Table 9: Metabolic products of different *E. coli* strains expressing the synthetic DHB pathway.

Genotype[1]	Plasmid[2]	DHB [g/l]	Acetate [g/l]	Cells [g/l]	Y_{DHB} [mol/mol]
wt	pHOM	0.00	2.18 ±0.12	3.59 ±0.04	0
wt	pDHB	0.06 ±0.01	3.04 ±0.25	3.63 ±0.17	0.004
ΔpoxB	pDHB	0.41 ±0.01	0	3.84 ±0.11	0.031
ΔackA-pta	pDHB	0.29 ±0.02	0.32 ±0.03	4.82 ±0.71	0.021
ΔackA-pta ΔpoxB	pDHB	0.27 ±0.04	0.05 ±0.01	4.54 ±0.59	0.021
wt	pACT3-ppc*	0.00	1.54 ±0.18	3.89 ±0.17	0
wt	pDHB-ppc*	1.07 ±0.02	0	3.47 ±0.12	0.09
**wt**	**pDHBopt-ppc***	**1.79 ±0.20**	**0**	**4.73** ±0.21	**0.15**
ΔpoxB	pDHBopt-ppc*	0.88 ±0.00	0	5.52 ±0.56	0.07
ΔackA-pta	pDHBopt-ppc*	1.60 ±0.06	0	5.56 ±0.22	0.12
ΔackA-pta ΔpoxB	pDHBopt-ppc*	1.26 ±0.01	0	5.24 ±0.34	0.10
wt	pDHBopt-ppc*(Ec-asd*)	0.74 ±0.39	0.25 ±0.11	4.33 ±0.66	0.06
wt	pDHBopt-ppc*(Mj-asd*)	0.77 ±0.17	0.28 ±0.05	4.13 ±0.41	0.06

Cells were cultivated in shake flasks on mineral M9 medium containing 20 g/l glucose. Values correspond to concentrations and yields after 24 h of cultivation. (1) All strains were derived from *E. coli* MG1655. (2) All plasmids were derived from the pACT3 medium-copy number plasmid. pHOM expresses the genes encoding the homoserine pathway enzymes Ec-LysC E250K, Ec-Asd, Sc-Hom6. pDHB expresses the genes encoding the DHB pathway enzymes MK: Ec-LysC V115A:E119S:E250K:E434V, MSD: Bs-Asd E218Q, MSR: Ms-Ssr H39R:N43H. pDHB-ppc* additionally expresses the malate-insensitive PEP carboxylase mutant Ppc_{K620S}. pDHBopt-ppc*has optimized ribosome binding sites in front of each DHB pathway gene. pDHBopt-ppc*(Ec-asd*) and pDHBopt-ppc*(Mj-asd*) express, respectively, the Ec-Asd_{E241Q} or Mj-Asd_{E210Q} mutant enzymes instead of Bs-Asd_{E218Q}. pACT3-ppc* only expresses Ppc_{K620S}.

Supplementary Table 10: Enzymatic activities of DHB pathway enzymes and PEP carboxylase

Enzymatic activity	Wild-type	Plasmid pDHB-ppc*	Plasmid pDHBop-ppc*
PEP carboxylase	0.53 ±0.02	2.9 ±0.14	2.5 ±0.79
Malate kinase	0	0.16 ±0.02	0.31 ±0.11
MSA dehydrogenase	0	bd$	0.012 ±0.004
MSA reductase	0	0.050 ±0.007	0.056 ±0.012

Results are presented as means ±SD of at least two biological replicate experiments. $bd : below detection

Supplementary Table 11: Primers and restriction enzymes used to clone genes into the pET28a expression vector.

Gene	Primers (5' – 3')	Restriction enzymes	Resulting vector
Ec-*lysC*	CACGAGGTACATATGTCTGAAATTGTTGTCTCC<br>CTTCCAGGGGATCCAGTATTTACTCAAAC	NdeI, *Bam*HI	pET28-Ec-lysC
Ec-*asd*	TATAATGCTAGCATGAAAAATGTTGGTTTTATCGG<br>TATAATGGA-TCCTTACGCCAGTTGACGAAGC	*Nhe*I, *Bam*HI	pET28-Ec-asd
Bs-*asd*	TATAATGCTAGCATGGGAAGAGGTTTACAC<br>TATAATGAATTCGCCTTACTCTCTTCTATTCGTC	*NheI, EcoRI*	pET28-Bs-asd
Mj-*asd*	Provided in vector by Eurofins	*NheI, EcoRI*	pET28-Mj-asd
Ms-*ssr*	TATAATGCTAGCATGAAAGCTGCAGTACTTCA<br>TATAATGAATTCTTACGGGATTATGAGACTTC	*Nhe*I, *Eco*RI	pET28-Ms-ssr
Sc-*YPR1*	TATAATGCTAGCATGCCTGCTACGTTAAAGAA<br>TATAATGAGCTCTCATTGGAAAATTGGGAAGG	*Nhe*I, *Sac*I	pET28-Sc-ypr1
Ec-*yqhD*	TATAATGAATTCTTAGCGGGCGGCTTCGTATATACGGCGGCTGACA<br>TATCGTGCTAGCATGAACAACTTTAATCTGCACA	*Nhe*I, *Eco*RI	pET28-Ec-yqhD
Pg-*4hbd*	TATAATGGATCCTTAGTAGAGTCTTCTGTAG<br>TATAATCATATGCAACTTTTCAAACTC	*Nde*I, *Bam*HI	pET28-Pg-4hbd

Ec- E. coli, Bs – B. subtilis, Mj – M. jannaschii, Sc- S. cerevisiae, Pg- P. gingivalis

Supplementary Table 12: Primers used for site-directed mutagenesis

Matrix	Mutated position	Primers (5' – 3')	Restriction site
pET28-Ec-lysC	E250K	GCGTTTGCCGAAGCGGCAAAGATGGCCACTTTTG<br>CAAAAGTGGCCATCTTTGCCGCTTCGGCAAACGC	*Eae*I
pET28-Ec-lysC	T344M	GGTAGATCTAATCACCATGTCAGAAGTGAGCGTGG<br>CCACGCTCACTTCTGACATGGTGATTAGATCTACC	none
pET28-Ec-lysC	T352I	GTCAGAAGTGAGCGTGGCATTAATTCTAGATACCAC<br>GTGGTATCTAGAATTAATGCCACGCTCACTTCTGAC	none
pET28-Ec-lysC	S345L	GGTAGATCTAATCACCACGTTAGAAGTGAGCGTGGC<br>GCCACGCTCACTTCTAACGTGGTGATTAGATCTACC	none
pET28-Ec-lysC	E119nnk	GCTGGTCAGCCATGGCNNKCTGATGTCGACCCTGC<br>GCAGGGTCGACATCAGMNNGCCATGGCTGACCAGC	*NcoI*
pET28-Ec-asd	E241nnk	AGCTCGATAACGGTCAGAGTCGANNKGAGTGGAAAGGGC AGGCGG<br>CCGCCTGCCCTTTCCACTCMNNTCGACTCTGACCGTTATCG AGCT	*Taq*I
pET28-Bs-asd	E218Q	AAATTCCAAGATAACGGCTATACGTTTCAGGAAATGAAAA TGATCAATG<br>CATTGATCATTTTCATTTCCTGAAACGTATAGCCGTTATCTT GGAATTT	none
pET28-Bs-asd	E218C	AAATTCCAAGATAACGGCTATACGTTTTGCGAAATGAAAAT GATCAATG<br>CATTGATCATTTTCATTTCGCAAAACGTATAGCCGTTATCTT GGAATTT	none
pET28-Mj-asd	E210Q	GATAACCTGATTCCCTTTATAAAGAATCAGGAAGAAAAGA TGCAGACCG<br>CGGTCTGCATCTTTTCTTCCTGATTCTTTATAAAGGGAATCA GGTTATC	none
pET28-Mj-asd	E210C	GATAACCTGATTCCCTTTATAAAGAATTGCGAAGAAAAGAT GCAGACCG<br>CGGTCTGCATCTTTTCTTCGCAATTCTTTATAAAGGGAATCA GGTTATC	none
pET28-Ms-ssr	H39R	GTCAAGGCAACCGGTCTCTGTCGCTCCGACGTCAATG<br>CATTGACGTCGGAGCGACAGAGACCGGTTGCCTTGAC	Nhe1
pET28-Ms-ssr	N43H	GGCTCTGTCACTCCGACGTACATGTCTTTGAGGGGAAAAC<br>GTTTTCCCCTCAAAGACATGTACGTCGGAGTGACAGAGCC	Nhe1

Supplementary Table 13: Primers used for LysC library construction

Primer	Sequence (5' – 3')
pETseq_for	ATGCGTCCGGCGTAGA
pETseq_rev	GCTAGTTATTGCTCAGCGG
lysC_A40A-T45S	GTGCGTTTAGTTGTCCTCTCGGCTTCTGCTGGGATCTCTAATCTGCTGGTCGCTTTAGCTG
lysC_A40S-T45T	GTGCGTTTAGTTGTCCTCTCGAGTTCTGCTGGGATCACTAATCTGCTGGTCGCTTTAGCTG
lysC_A40S-T45S	GTGCGTTTAGTTGTCCTCTCGAGTTCTGCTGGGATCTCTAATCTGCTGGTCGCTTTAGCTG
lysC_V115S-	CTGACAGATGAGCTGAGCAGCCATGGCGATCTGATGTCGACCCTG
lysC_V115S-	CTGACAGATGAGCTGAGCAGCCATGGCGGGCTGATGTCGACCCTG
lysC_V115S-	CTGACAGATGAGCTGAGCAGCCATGGCAATCTGATGTCGACCCTG
lysC_V115S-	CTGACAGATGAGCTGAGCAGCCATGGCCAGCTGATGTCGACCCTG
lysC_V115S-	CTGACAGATGAGCTGAGCAGCCATGGCTCGCTGATGTCGACCCTG
lysC_V115A-	CTGACAGATGAGCTGGCCAGCCATGGCGATCTGATGTCGACCCTG
lysC_V115A-	CTGACAGATGAGCTGGCCAGCCATGGCGGCCTGATGTCGACCCTG
lysC_V115A-	CTGACAGATGAGCTGGCCAGCCATGGCAATCTGATGTCGACCCTG
lysC_V115A-	CTGACAGATGAGCTGGCCAGCCATGGCCAGCTGATGTCGACCCTG
lysC_V115A-	CTGACAGATGAGCTGGCCAGCCATGGCTCGCTGATGTCGACCCTG
lysC_V115V-	CTGACAGATGAGCTGGTCAGCCATGGCGATCTGATGTCGACCCTG
lysC_V115V-	CTGACAGATGAGCTGGTCAGCCATGGCGGGCTGATGTCGACCCTG
lysC_V115V-	CTGACAGATGAGCTGGTCAGCCATGGCAATCTGATGTCGACCCTG
lysC_V115V-	CTGACAGATGAGCTGGTCAGCCATGGCCAGCTGATGTCGACCCTG
lysC_V115V-	CTGACAGATGAGCTGGTCAGCCATGGCTCGCTGATGTCGACCCTG
lysC_F184G	GCTTAGTTATAACCCAGGGAGGTATCGGTAGCGAAAATAAAG
lysC_T195R	AAAATAAAGGTCGTACAACGAGGCTTGGCCGCGG
lysC_T195S	AAAATAAAGGTCGTACAACGTCGCTTGGCCGCGG
lysC_S201A	CTTGGCCGTGGAGGCGCCGATTATACGGCAGC
lysC_T359D	CTTGATACCACAGGTTCAGACTCCACTGGCGATACGTT
lysC_T359V	CTTGATACCACAGGTTCAGTCTCCACTGGCGATACGTT
Ec_lysC_clon_fo	CACGAGGTACATATGTCTGAAATTGTTGTCTCC
Ec_lysC_clon_re	CTTCCAGGGGATCCAGTATTTACTCAAAC

Supplementary Table 14: Plasmids used and constructed in this study

Name	Relevant characteristics	Reference
Plasmids		
pKD4	Amp^R, FRT-*kan*-FRT, temperature-sensitive replicon	[10]
pKD46	Amp^R, harbors λ, β, exo (λ-Red recombinase), temperature-sensitive replicon	[10]
pCP20	Amp^R, Cm^R, harbors FLP recombinase, temperature-sensitive replicon	[11]
pACT3	Cm^R, p15A origin, tac promoter	[7]
pACT3-ppc	pACT3 derivative harboring *Ec-ppc*	This work
pACT3-ppc*	pACT3 derivative harboring $Ec\text{-}ppc_{K620S}$	This work
pHOM	pACT3 derivative harboring $Ec\text{-}lysC_{E250K}$, *Ec-asd, Sc-HOM6*	[12]
pDHB	pACT3 derivative harboring $Ec\text{-}lysC_{V115A:E119S:E250K:E343V}$, $Bs\text{-}asd_{E218Q}$, $Ms\text{-}Ssr_{H39R\ N43H}$	This work
pDHB-ppc*	pDHB derivative additionally harboring $Ec\text{-}ppc_{K620S}$	This work
pDHBop-ppc*	pDHB-ppc* with optimised RBS sequences in front of DHB pathway genes	This work
pDHBop(Mj-asd*)-ppc*	pDHBop-ppc* derivative harboring $Mj\text{-}asd_{E210Q}$instead of $Bs\text{-}asd_{E218Q}$	This work
pDHBop(Ec-asd*)-ppc*	pDHBop-ppc* derivative harboring $Ec\text{-}asd_{E241Q}$instead of $Bs\text{-}asd_{E218Q}$	This work

Supplementary Table 15: Primers used for assembly of the operons.

Primer	Sequence (5' – 3')*
ppc_clon_for	TATAATCCCGGGATGAACGAACAATATTCC
ppc_clon_rev	TATAATTCTAGATTAGCCGGTATTACGCAT
ppc_k620s_for	CGCTTTAGCTATGGTCTGCCAGAAATCACCGAG
ppc_k620s_rev	CCATAGCTAAAGCGGATCATCTCGCCC
lysC-IF-forward	CAGGAAACAGAATTCGAGCTCTCTAGATGGAAGGAGGCAGCCATATGTCTGAAATTGTTGTCTCC
lysC-IF-reverse	ACTCCTTCGGATCCTTAAACGGATCCAGTATTTACTCAAACAAATTACTATGC
asd-IF-forward	TTTAAGGATCCGAAGGAGTGGCTAGCATGCATGGGAAGAGGTTTACAC
asd-IF-reverse	ACTCCTTCAGATCTACGGAGCTCGAATTCTTACTCTCTTCTATTCGTC
IF_ppc_lysC_For	TAATACCGGCTAATCTAGAGAAGGAGGCAGCCATATGTCTGAAATTG
IF_pACT_ssr_Rev	ATCCGCCAAAACAGAAGCTTCTCGAGCTTACGGAATAATCAGGCTACG
ssr-IF-forward	TAGATCTGAAGGAGTGGCTAGCATGAAAGCAGCA
ssr-IF-reverse	ATCCGCCAAAACAGAAGCTTGTCTCGAGCTTACGGAATAATCAGGCTAC
lysC-rbs-forward	TATAAATCTAGAACTATAAGAATAAAAAATTATAAGGAGGTTTAAAAATGTCTGAAATTGTTGTCTCCAAATT
lysC-rbs-reverse	CGACAACAGCTACGTGTAAACCTCTTCCCATATAATACCTCCTTACTTTGTGTGTTCCTTGACTAGTTTACTCAAACAAATTACTATGCAGTTTTTG
asd-rbs-forward	ATGGGAAGAGGTTTACACGTAGCT
asd-rbs-reverse	TATAAACTTAAGATATAGATCTTTATACGAGGTTTAGTTTTTTCAGGCTT
ssr-rbs-forward	TATAAGATCTGAGACAAGGAATTAACGTAAATAAGGAGGTATAGCATGAAAGCAGCAGTTCTGC
ssr-rbs-reverse	TATAACTCGAGCTTACGGAATAATCAGGCTACGA
Mj-asd_clon_for	ACTAGTCAAGGAACACACAAAGTAAGGAGGTATTATATGAGCAAAGGGGAGAAAATG
Mj-asd_clon_rev	TAAGATCTTTAAATATATTTCTTCACGAAGTATTCCGC
Ec-asd_clon_for	ACTAGTCAAGGAACACACAAAGTAAGGAGGTATTATATGAAAAATGTTGGTTTTATCGGC
Ec-asd_clon_rev	TAAGATCTTTAAATATATTTCTTCACGAAGTATTCCGC

(*) Underlined sequences correspond to the restriction sites mentioned in the text.

Supplementary Table 16: Primers used for strain constructions

Primer	Sequence (5' – 3')
Delta_ackA/pta_for	ATGTCGAGTAAGTTAGTACTGGTTCTGAACTGCGGTAGTTCTTCAGTGTAGGCTGGAGCTGCTTC
Delta_ackA/pta_rev	TTACTGCTGCTGTGCAGACTGAATCGCAGTCAGCGCGATGGTGTACATATGAATATCCTCCTTAG
ackA/pta_locus_f	CCATTGGCTGAAAATTACGC
ackA/pta_locus_r	TCCCTTGCACAAAACAAAGT
poxB_locus_f	CGATGATATTCCTTTCATCGGGC
poxB_locus_r	CGTAAATCAATCATGGCATGTCC

Supplementary Table 17: Strains used in this study

Strain reference	Genotype	Reference
MG1655	$F^- \lambda^-$ ilvG- rfb-50 rph-1	ATCC 47076
NEB5-α	*fhuA2 Δ(argF-lacZ)U169 phoA glnV44 Φ80Δ (lacZ)M15 gyrA96 recA1 relA1 endA1 thi-1 hsdR17*	NEB
JW0855-1	F-*, Δ(araD-araB)567, ΔlacZ4787(::rrnB-3), λ-, ΔpoxB772::kan, rph-1, Δ(rhaD-rhaB)568, hsdR514*	[14]
	MG1655 harboring pHOM	This study
	MG1655 harboring pDHB	This study
	ΔackA-pta::FRT harboring pDHB	This study
	ΔpoxB::FRT harboring pDHB	This study
	ΔackA-pta::FRT*ΔpoxB::FRT* harboring pDHB	This study
	MG1655 harboring pACT3-ppc*	This study
	MG1655 harboring pDHBop-ppc*	This study
	ΔackA-pta::FRT harboring pDHBop-ppc*	This study
	ΔpoxB::FRT harboring pDHBop-ppc*	This study
	ΔackA-pta::FRT*ΔpoxB::FRT* harboring pDHBop-ppc*	This study
	MG1655 harboring pDHBop(Mj-asd*)-ppc*	This study
	MG1655 harboring pDHBop(Ec-asd*)-ppc*	This study

Supplementary Note 1: *Standard Gibbs free energy of the DHB pathway*

The stoichiometry of the reactions malate kinase (MK), malate semialdehyde dehydrogenase (MSD) and malate semialdehyde reductase (MSR) is as follows (Equations S1 – S3):

MK: malate^{2-} + ATP^{3-} + H^{+} = malyl-P^{2-} + ADP^{2-} (S1)

MSD: malyl-P^{2-} + NADPH = malate-SA^{-} + NADP^{+} + HPO_4^{2-} (S2)

MSR: malate-SA^{-} + NADPH + H^{+} = DHB^{-} + NADP^{+} (S3)

The standard Gibbs free energy for the formation of the DHB pathway intermediates ($\Delta_f G^{0\prime}$) was calculated based on the group contribution theory[1] using the dataset published by Jankowski[2] (Supplementary Table 1).

The standard Gibbs free energy of a reaction ($\Delta_r G^{0\prime}$) can be estimated according to Equation S4 from the standard Gibbs free energy of the formation of the participating compounds ($\Delta_f G^{0\prime}$), with v_i being the stoichiometric coefficients.

$$\Delta_r G^{0\prime} = \sum_i v_i \cdot \Delta_f G_i^{0\prime} \qquad \text{(S4)}$$

Accordingly, the standard Gibbs free energy of the reactions MK, MSD, MSR, and the entire pathway are, respectively, 11 kJ mol^{-1}, 1.5 kJ mol^{-1}, -21.8 kJ mol^{-1} and -9.3 kJ mol^{-1}.

Supplementary Note 2: *Calculation of the theoretical 2,4-dihydroxybutyric acid yield*

A previously published stoichiometric model of the central carbon metabolism in *E. coli*[3] was extended by the 3 DHB pathway reactions. The theoretical yield was calculated based on elementary mode analysis using the CellNetAnalyzer software package[4]. The flux map showing one of the predicted carbon flux distributions that provide maximum yield is depicted in Supplementary Figure 1. In the absence of cell growth the maximum DHB yield is 1.5 mol per mol glucose.

Supplementary Note 3: *Enzymatic activity of wild-type homoserine pathway enzymes on synthetic DHB pathway intermediates*

The homoserine pathway in *E. coli* is in part catalysed by two bi-functional enzymes, namely ThrA and MetL, which both possess aspartate kinase (AK) and homoserine dehydrogenase (HSD) activity[5,6]. To facilitate enzyme engineering we decided to work only with mono-functional enzymes and therefore tested the wild-type *E. coli* enzymes AK III, encoded by *lysC*[7], and aspartate semialdehyde

dehydrogenase (ASD), encoded by *asd*[8], as well as the mono-functional HSD from *Saccharomyces cerevisiae*, encoded by *HOM6*[9], for their ability to accept the corresponding DHB pathway intermediates as substrates. As reported in Supplementary Table 2, these enzymes displayed almost no activity on the synthetic substrate of the DHB pathway.

Supplementary Note 4: *Structural analysis of EC-LysC and construction of mutant library for the engineering of malate kinase activity*

Several factors collectively suggest that catalytically productive binding of (L)-malate may be sterically compromised in the absence of a strong favourable electrostatic interaction with the glutamate side-chain carboxylate group at position 119 in the enzyme. These include an earlier report that (L)-malate is a weak competitive inhibitor of Ec-LysC activity on (L)-aspartate[11], the observed sluggish kinetic behaviour of the Ec-LysC E119Q variant towards (L)-malate (Supplementary Table 3), and the demonstration of improved catalytic performance in mutants containing smaller glycine, alanine, serine or cysteine amino acid replacements of Glu119 (Supplementary Table 3). We thus sought to optimise (L)-malate substrate binding interaction complementarity by experimental screening of a designed Ec-LysC mutant library. The identification of target residue positions and candidate replacement residue types for construction of the library was guided by an analysis of natural sequence variation and tolerance to mutation in aspartate kinases, and the computational re-design of amino acid residues at positions within the active-site region.

A multiple sequence alignment of 1000 Ec-LysC homologues, identified by BLAST searching of the non-redundant NCBI sequence database, was created using MUSCLE[12]. Analysis of the multiple sequence alignment, summarised in Supplementary Table 4, showed residue positions occupied by Glu119, Arg198 and Thr45 to be entirely conserved. Ser201 (95.2%) and Phe184 (93.7%) are also heavily conserved, and essentially subject only to conservative replacement by Thr (4.1 %) and Tyr (6.3 %), respectively.

Normalised values of the Shannon entropy (H_X), calculated from residue type variation profiles in the multiple sequence alignment as described in Methods (Computational Methods section), were used as a metric of position (X)-dependent residue variation. H_X is zero for a completely conserved residue position, and has a maximum value of 1, corresponding to a distribution in which each residue type has an equal probability of occurrence. H_X values of 0.33 were observed at residue positions 39 and 40 that show the highest tolerance to residue type variation amongst the substrate contacting residues in the first binding shell. Alanine substitution by serine was observed at residue position 40 in the multiple sequence alignment. Although the methyl group of Ala40 was observed to be too far away to make direct contact with a bound (L)-malate molecule in

a modelled complex with Ec-LysC, residue substitution by serine appeared able to permit the introduction of a side-chain hydrogen bond with the (L)-malate 2-OH hydroxyl group either directly, or indirectly through the mediation of a hydrogen bond to a side-chain oxygen atom in a glutamate or a glutamine residue at position 119.

Three other residue positions (Val115, Thr195 and Thr359) with elevated values of H_X in the range 0.47 to 0.82 were located within the second shell of active-site residues that do not make direct contact with the substrate (Figure 2A and Supplementary Figure 2). Contacting side-chain atoms of Phe184 contribute significantly to occluded surfaces[13] of the Val115, Thr195 and Thr359 side-chain atoms. It was reasoned that replacement of the bulky aromatic Phe184 residue by a smaller residue could create sufficient space to permit the incorporation of larger side-chains at second-shell positions 115, 195 or 359 able to contact the (L)-malate substrate directly.

Computational re-design was performed at nine first and second shell residue positions (Supplementary Table 4) in the active-site region of a starting model of a binary complex of the E119Q mutant with (L)-malate using RosettaDesign software[14] as described in Methods. Residue type frequency distributions at each residue position in solutions of lowest energy are summarised below in Supplementary Figure 3.

The results identified between one and three different residue types at eight positions, with only Ser39 in the starting sequence left unchanged. Phe184 which exists in a positive ϕ main-chain conformation was replaced in all solutions by glycine. Ala40 was systematically replaced by serine. The most variation was observed at the second shell 115, 195 or 359 residue positions. The glutamine residue, present at position 119 in the initial starting enzyme structure, was systematically replaced by aspartate.

The eight residue positions at which non-native residue types were identified by computational re-design were included as target positions in the design of the mutant library (Supplementary Table 5). All non-native residue types present at these positions in computational solutions of lowest energy were permitted in the combinatorial library. Glycine, serine, asparagine and glutamine residue types displaying enzyme activity towards (L)-malate in Glu119 point site mutants (Supplementary Table 3) were additionally allowed at position 119. Finally, the library was supplemented by native amino acid residues in Ec-LysC that were not recovered by computational re-design (Ala40, Thr45 and Phe184).

Supplementary Note 5*: Engineering of malate semialdehyde reductase activity*

A set of enzymes with reported activities on aldehydes that are structurally cognate to malate semialdehyde was tested for activity on this DHB pathway intermediate. We chose to test the broad range aldehyde reductase, YqhD[15], and both tartronyl semialdehyde reductases, GarR[16] and GlxR[17], from *E. coli*; the methyl-butyraldehyde reductase, Ypr1[18], and the medium-chain alcohol dehydrogenase, Adh6[19], from *Saccharomyces cerevisiae*; the succinic semialdehyde reductase, Ssr, from *Metallosphaera sedula*[20], and the 4-hydroxybutyrate dehydrogenase, 4hbd[21,22], from *Porphyromonas gingivalis*. The succinic semialdehyde reductase from *M. sedula* (Ms-Ssr) had the highest activity with a k_{cat}/Km value of 5.1 s^{-1} mM^{-1} (Supplementary Table 7).

Supplementary References

1. Mavrovouniotis, M. L. Estimation of standard Gibbs energy changes of biotransformations. *J. Biol. Chem.* **266,** 14440–14445 (1991).

2. Jankowski, M. D., Henry, C. S., Broadbelt, L. J. & Hatzimanikatis, V. Group contribution method for thermodynamic analysis of complex metabolic networks. *Biophys. J.* **95,** 1487–1499 (2008).

3. Stelling, J., Klamt, S., Bettenbrock, K., Schuster, S. & Gilles, E. D. Metabolic network structure determines key aspects of functionality and regulation. *Nature* **420,** 190–193 (2002).

4. Klamt, S., Saez-Rodriguez, J. & Gilles, E. D. Structural and functional analysis of cellular networks with CellNetAnalyzer. *BMC Syst. Biol.* **1,** 2 (2007).

5. Falcoz-Kelly, F., van Rapenbusch, R. & Cohen, G. N. The methionine-repressible homoserine dehydrogenase and aspartokinase activities of *Escherichia coli* K 12. Preparation of the homogeneous protein catalyzing the two activities. Molecular weight of the native enzyme and of its subunits. *Eur. J. Biochem.* **8,** 146–152 (1969).

6. Starnes, W. L. *et al.* Threonine-sensitive aspartokinase-homoserine dehydrogenase complex, amino acid composition, molecular weight, and subunit composition of the complex. *Biochemistry* **11,** 677–687 (1972).

7. Cassan, M., Parsot, C., Cohen, G. N. & Patte, J. C. Nucleotide sequence of *lysC* gene encoding the lysine-sensitive aspartokinase III of *Escherichia coli* K12. Evolutionary pathway leading to three isofunctional enzymes. *J. Biol. Chem.* **261,** 1052–1057 (1986).

8. Haziza, C., Stragier, P. & Patte, J. C. Nucleotide sequence of the asd gene of *Escherichia coli*: absence of a typical attenuation signal. *EMBO J.* **1,** 379–384 (1982).

9. Thomas, D., Barbey, R. & Surdin-Kerjan, Y. Evolutionary relationships between yeast and bacterial homoserine dehydrogenases. *FEBS Lett.* **323,** 289–293 (1993).

10. Kotaka, M., Ren, J., Lockyer, M., Hawkins, A. R. & Stammers, D. K. Structures of R- and T-state *Escherichia coli* aspartokinase III. Mechanisms of the allosteric transition and inhibition by lysine. *J. Biol. Chem.* **281,** 31544–31552 (2006).

11. Keng, Y. F. & Viola, R. E. Specificity of aspartokinase III from *Escherichia coli* and an examination of important catalytic residues. *Arch. Biochem. Biophys.* **335,** 73–81 (1996).

12. Edgar, R. C. MUSCLE: multiple sequence alignment with high accuracy and high throughput. *Nucleic Acids Res.* **32,** 1792–1797 (2004).

13. Pattabiraman, N., Ward, K. B. & Fleming, P. J. Occluded molecular surface: analysis of protein packing. *J. Mol. Recognit.* **8,** 334–344 (1995).

14. Leaver-Fay, A. *et al.* ROSETTA3: an object-oriented software suite for the simulation and design of macromolecules. *Methods Enzymol.* **487,** 545–574 (2011).

15. Jarboe, L. R. YqhD: a broad-substrate range aldehyde reductase with various applications in production of biorenewable fuels and chemicals. *Appl. Microbiol. Biotechnol.* **89,** 249–257 (2011).

16. Hubbard, B. K., Koch, M., Palmer, D. R., Babbitt, P. C. & Gerlt, J. A. Evolution of enzymatic activities in the enolase superfamily: characterization of the (D)-glucarate/galactarate catabolic pathway in *Escherichia coli*. *Biochemistry* **37,** 14369–14375 (1998).

17. Njau, R. K., Herndon, C. A. & Hawes, J. W. Novel beta-hydroxyacid dehydrogenases in *Escherichia coli* and *Haemophilus influenzae*. *J. Biol. Chem.* **275,** 38780–38786 (2000).

18. Ford, G. & Ellis, E. M. Characterization of Ypr1p from *Saccharomyces cerevisiae* as a 2-methylbutyraldehyde reductase. *Yeast* **19,** 1087–1096 (2002).

19. Larroy, C., Fernández, M. R., González, E., Parés, X. & Biosca, J. A. Characterization of the *Saccharomyces cerevisiae* YMR318C (ADH6) gene product as a broad specificity NADPH-dependent alcohol dehydrogenase: relevance in aldehyde reduction. *Biochem. J.* **361,** 163–172 (2002).

20. Kockelkorn, D. & Fuchs, G. Malonic semialdehyde reductase, succinic semialdehyde reductase, and succinyl-coenzyme A reductase from Metallosphaera sedula: enzymes of the autotrophic 3-hydroxypropionate/4-hydroxybutyrate cycle in *Sulfolobales*. *J. Bacteriol.* **191,** 6352–6362 (2009).

21. Söhling, B. & Gottschalk, G. Molecular analysis of the anaerobic succinate degradation pathway in *Clostridium kluyveri*. *J. Bacteriol.* **178,** 871–880 (1996).

22. Nelson, K. E. *et al.* Complete genome sequence of the oral pathogenic bacterium *Porphyromonas gingivalis* strain W83. *J. Bacteriol.* **185,** 5591–5601 (2003).
